# Risk associated with central catheters for malignant tumor patients: a systematic review and meta-analysis

**DOI:** 10.18632/oncotarget.24212

**Published:** 2018-01-12

**Authors:** Yajuan Lv, Yong Hou, Bo Pan, Yuwan Ma, Paiyun Li, Lili Yu, Deguo Xu, Juanjuan Song, Heli Shang, Hongyan Wang, Yuan Tian

**Affiliations:** ^1^ Department of Radiation Oncology, Shandong Provincial Qianfoshan Hospital Affiliated to Shandong University, Jinan, Shandong, 250014, P.R. China; ^2^ Key Laboratory of Translational Research, Peking University Cancer Hospital and Institute, Beijing, 100142, China

**Keywords:** central catheters, PICC, CICC, thrombosis, mortality

## Abstract

The risk of venous thrombosis and mortality associated with central catheter (PICC/CICC) for malignant tumor patients is not definite. So, we carried out a systematic review and meta-analysis to evaluate it. Among patients with comparing PICC with CICC, odds ratio (OR) or risk ratio (RR) was calculated with a random effect model meta-analysis. The result of the stratification analysis of 7 studies (PICC vs CICC) supported the theory that CICCs were associated with a decrease in the odds ratio of thrombosis compared with PICCs. 7 of 15 studies provided the information about the compared mortality rate of the patients. The result showed that CICCs were associated with a decrease in the odds ratio of thrombosis compared with PICCs (OR = 0.45, 95% CI:0.32–0.62, *p* < 0.0001, I^2^ = 0%,Tau^2^ = 0.00). Meta-analysis of 8 studies of 2639 patients showed that pharmacological deep vein thrombosis prophylaxis drugs could decrease the risk of mortality of malignant tumor patients with CICCs (RR = 0.58, 95% CI:0.48–0.71, *Z* = 5.32, *p* < 0.0001, I^2^ = 71%). We found that PICCs are associated with a raised risk of deep vein thrombosis, and pharmacological deep vein thrombosis prophylaxis drugs is a beneficial factor in decreasing the incidence of thrombosis, while warfarin may decrease the risk of mortality of malignant tumor patients with CICCs.

## INTRODUCTION

Central catheter, including peripherally inserted central catheter (PICC) or central inserted central catheter (CICC), is a device used for many functions among cancer patients, including monitoring haemodynamic indicators and administering intravenous medications, fluids, blood products and parenteral nutrition. The use of it has increased rapidly, especially for PICC. Furthermore, nurse-leading PICC teams have made their use convenient and accessible in many settings [1, 2]. However, as a foreign inserted object, it is susceptible to deep vein thrombosis and pulmonary embolism which may increase cost, morbidity and mortality [3–5]. There were plenty of clinical trials about catheter-related infection or thrombosis, and we took a lot of measures to decrease the incidence rate of them among different patients [6–8]. However, the understanding of risk about central venous catheter-related side effects is still an important safety question to be resolved, especially for cancer patients as they are prone to be associated with a higher risk of deep vein thrombosis than others [9]. To our known, at present, there was no systematic review done to deal with these problems just limited to malignant tumor patients. Therefore, we carried out the systematic review and meta-analysis to further investigate this risk in malignant tumor patients and tried to reveal the relationship between central venous catheter-related side effects and them. We pay our attention mainly to catheter-related venous thrombosis, mortality and mitigation methods, and then compare the risk between PICC and CICC.

## RESULTS

**Figure 1:**
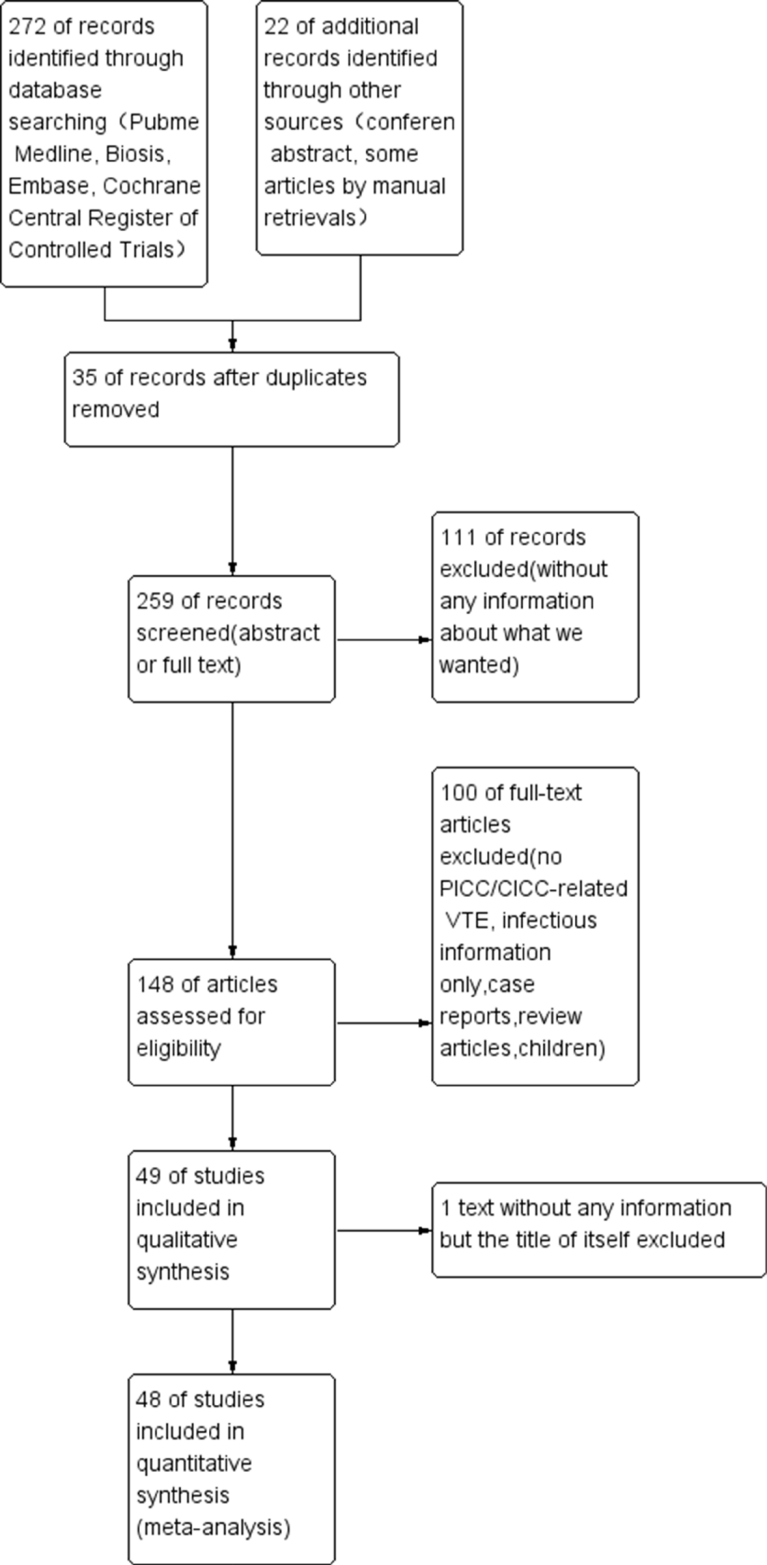
PRISMA flow diagram of our study

**Figure 2:**
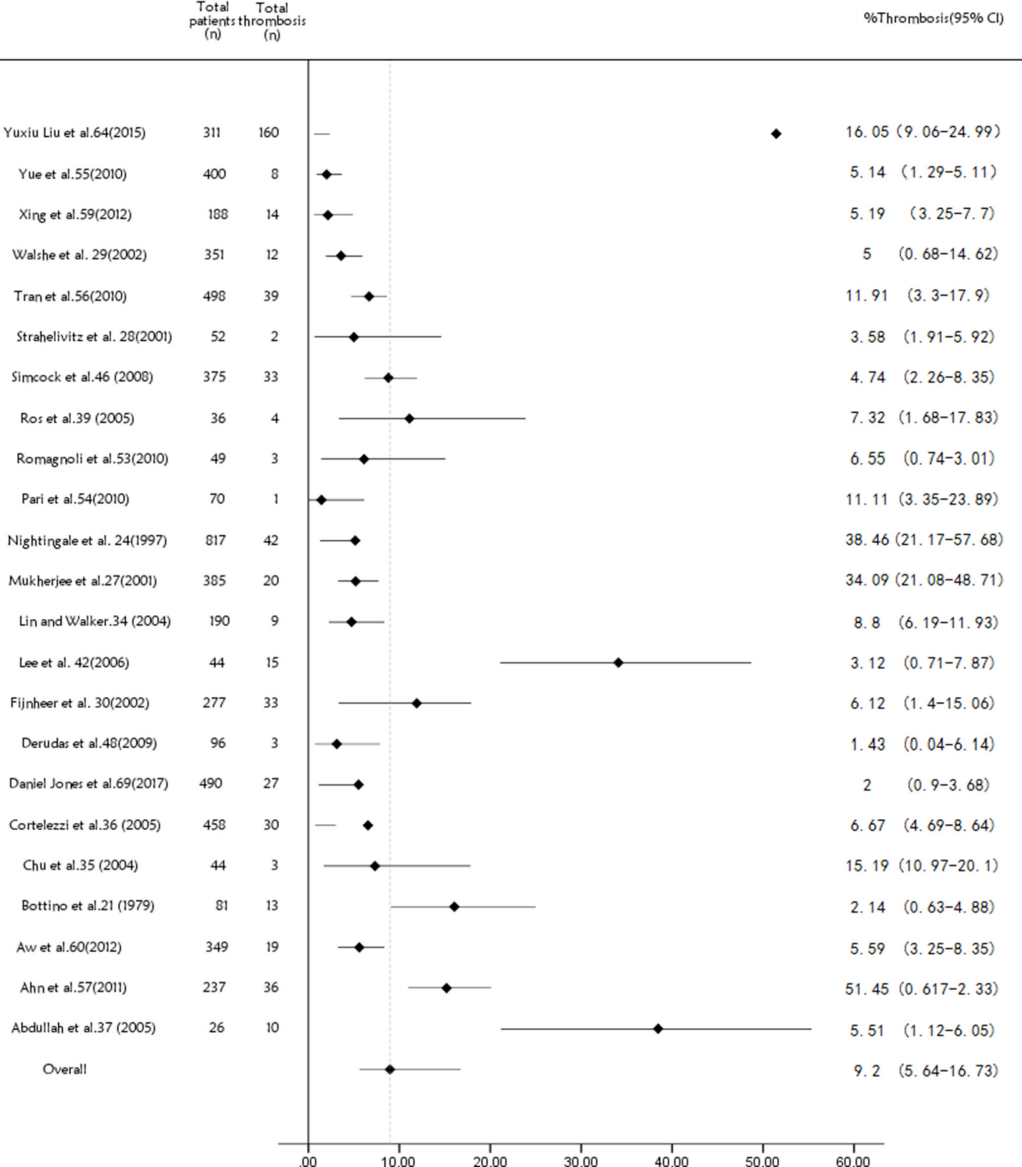
Forest plot and funnel plot showing the pooled, weighted frequency of patients with peripherally inserted central catheter VTE in studies without a comparison group PICC = peripherally inserted central catheter; CICC = central inserted central catheter; FE = fixed effect; RE = random effect; OR = odds ratio; RR = risk ratio; VTE = venous thromboembolism.

There were 294 articles and conference abstracts collected by us, the process of our search was shown in (Figure 1). Among all the citations identified by our electronic and manual searches, 148 articles met the preliminary inclusion criteria. Then, we imported summary of all the articles into EndNote X8, and red all the abstracts of them to make sure whether they were up to the standard criteria. Thus, 48 articles including 15508 central catheters fulfilled the eligibility criteria [21–68]. The characteristics of them were listed in (Supplementary Table 3). 7 studies compared PICC with CICC or Port (*n* = 2872) [22, 51, 57, 61, 62, 66, 67], only 1 study involving mortality information [61], whereas 23 included studies which just displayed PICC without a comparison group (*n* = 5824) [21, 23, 26–29, 33–36, 38, 41, 45, 47, 52–56, 58, 59, 63, 68]. 15 articles (*n* = 6579) showed the details of CICC with pharmacological deep venous thrombosis (DVT) prophylaxis compared with placebo or other drugs, such as heparin drugs, warfarin and other thrombolytic drugs [24, 25, 30–32, 37, 39, 40, 42–44, 46, 49, 50, 60]. 7 of 15 studies with pharmacological deep vein thrombosis prophylaxis data provided the information about the compared mortality rate of the patients, including cancer patients, haematological malignancies and a mixture of the two [30, 31, 39, 40, 42, 43, 49]. We could find 34 full text of the 48 studies, which were also marked in (Supplementary Table 3). The remainders were abstracts presented online or at some conference reports [22, 26, 32–34, 38, 45, 47, 48, 52–54, 56]. All the deep vein thrombosis, involved in every study, were confirmed by ultrasonography, X-ray, or CT scan. The maintenance of inserted catheters were listed in the (Supplementary Table 3). If it was marked as “NR” [22, 26–28, 33, 34, 38, 43, 45, 47, 52–60, 62, 66, 68], it means that they were maintained according to the catheter specifications without special medication. 23 non-comparison studies about PICC were just displayed by a forest plot showing the pooled, unweighted frequency of patients with peripherally inserted central catheter related venous thromboembolism (Figure 2). In these studies, the unweighted frequency of vein thrombosis was 9.2% (536/5824), which is higher than the former reported result 4.48% (189/4223) related to non-tumor and tumor mixed populations [9].

**Figure 3:**
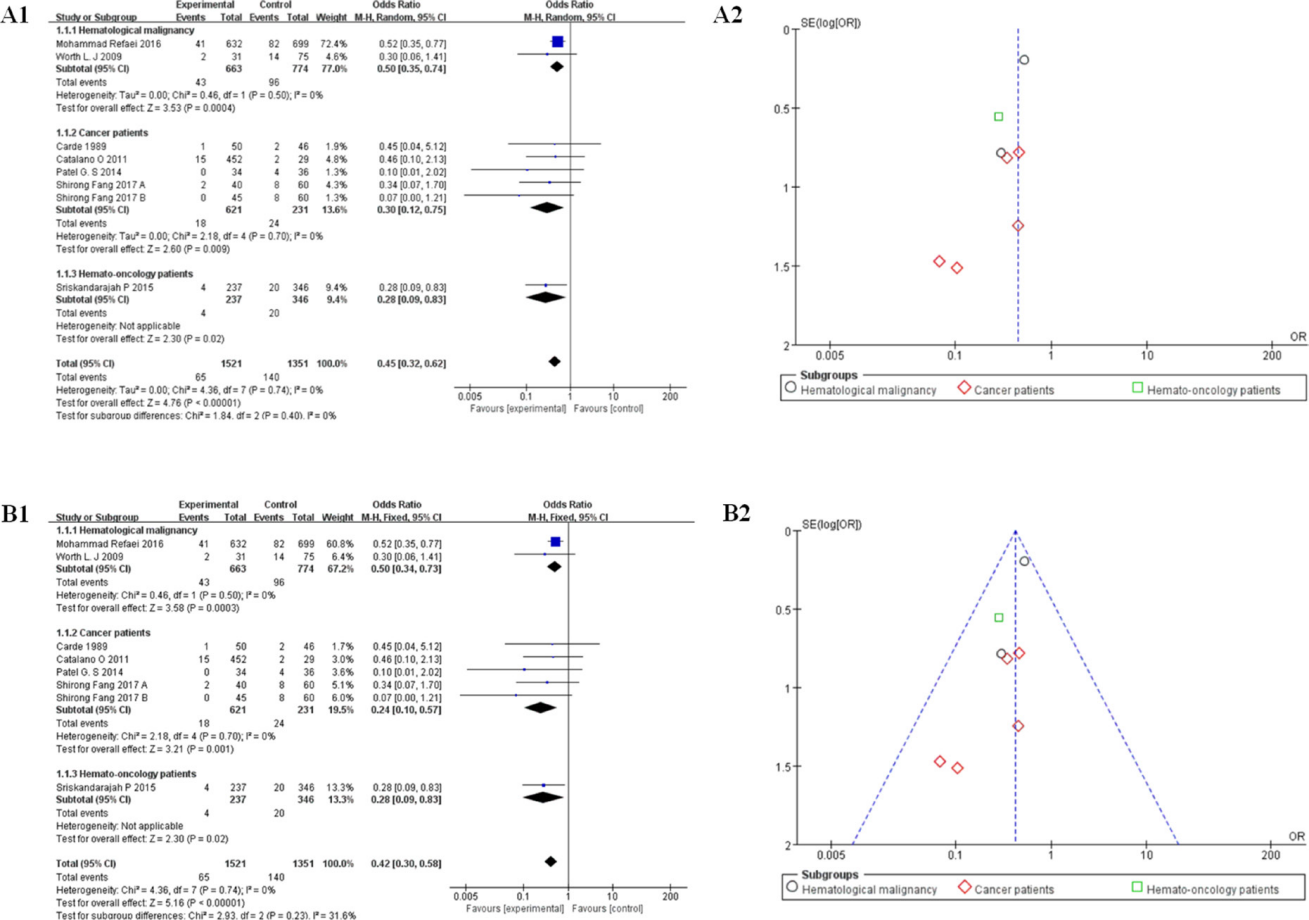
(**A**) Forest plot (A11,RE) and funnel plot (A2,RE) showing risk of venous thromboembolism between peripherally inserted central catheters (PICCs) and central inserted central catheters (CICCs) group. Forest plot showing odds ratio (OR) of development of upper-extremity DVT in patients with peripherally inserted central catheters versus central venous catheters. (**B**) Forest plot (B1, FE) and funnel plot (B2, FE) showing risk of venous thromboembolism between peripherally inserted central catheters (PICCs) and central inserted central catheters (CICCs) group. Forest plot showing odds ratio (OR) of development of upper-extremity DVT in patients with peripherally inserted central catheters versus central venous catheters. PICC = peripherally inserted central catheter; CICC = central inserted central catheter; FE = fixed effect; RE= random effect; OR = odds ratio; RR = risk ratio; VTE = venous thromboembolism.

7 studies (*n* = 2872) reported PICC-related versus CICC or Port-related vein thrombosis outcomes, which were divided into 3 parts for stratification analysis, listed as cancer patients [22, 57, 61, 67], hematological malignancies [51, 66], and hemato-oncology patients [62]. The unweighted frequency of deep vein thrombosis was 7.14% (205/2872) among them. We took a random effect model of meta-analysis to make stratification analysis. The result of it showed that CICCs were associated with a decrease in the odds of thrombosis compared with PICCs (OR = 0.45, 95% CI:0.32–0.62, *p* < 0.0001, I^2^ = 0%, Tau^2^ = 0.00; Figure 3A1), especially in cancer patients (OR = 0.30, 95% CI:0.12–0.75, *p* < 0.05, I^2^ = 0%, Tau^2^ = 0.00) [22, 57, 61, 67]. Based on the above results (I^2^ = 0%), we used the fixed model to analyze the raw data of 7 studies again. Similar overall results were shown among all the patients (OR = 0.42, 95% CI:0.30–0.58, *p* < 0.0001, I^2^ = 0%, Tau^2^ = 0.00, Figure 3B1), the stratification results of CICCs compared with PICCs in cancer patients showed a much more pronounced difference (OR = 0.24, 95% CI:0.10–0.57, I^2^ = 0%, *p* = 0.001). 3 of 7 studies referred to the maintenance methods of catheters without pharmacological deep venous thrombosis (DVT) prophylaxis information [51, 61, 67]. We gave up analyzing the patient’s death data because only 1 study included this information [61].

**Figure 4:**
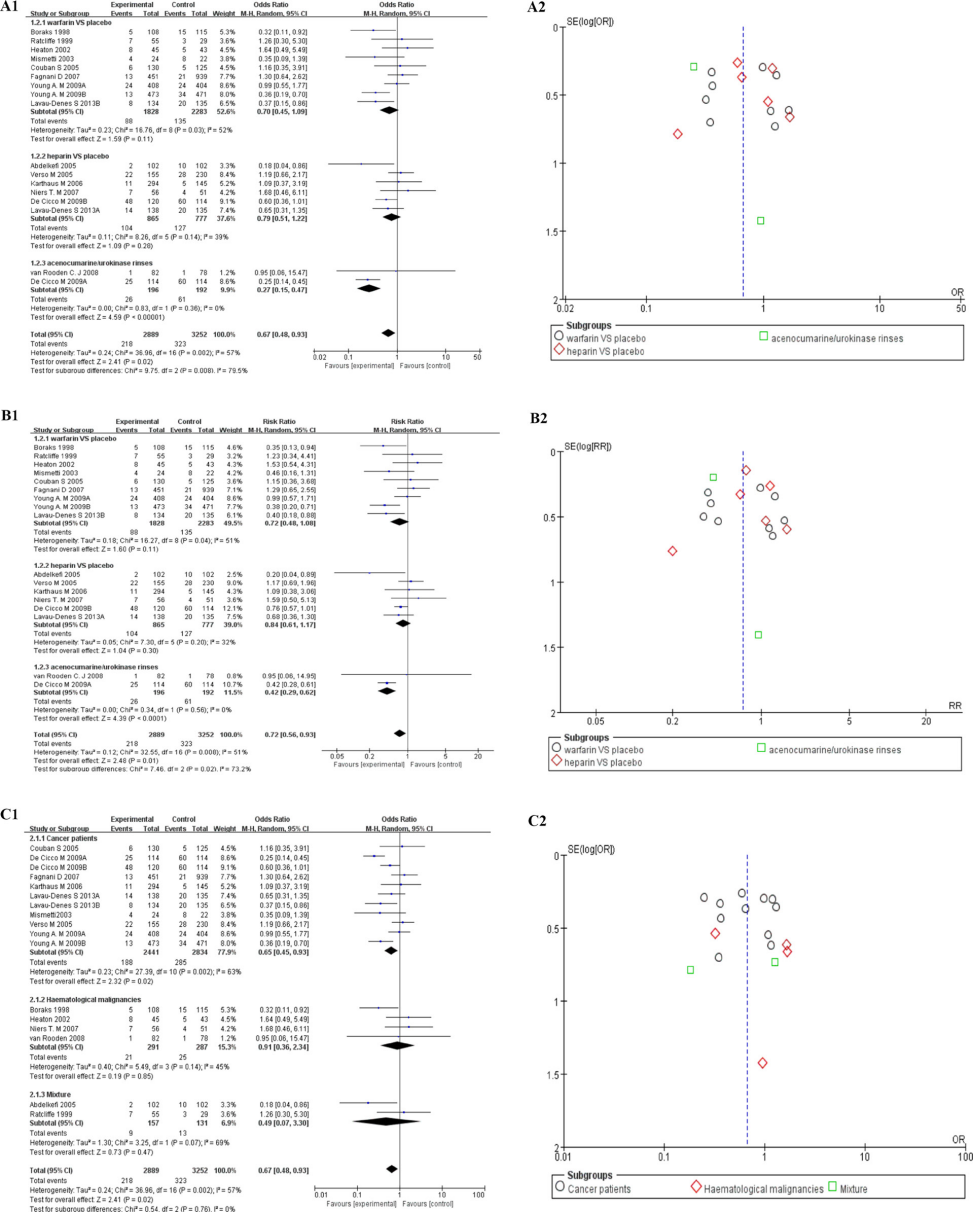
(**A**) Forest plot (A1, RE) and funnel plot (A2, RE) showing 15 studies of CICCs with pharmacological deep vein thrombosis prophylaxis data, stratified by anticoagulant drugs Forest plot showing odds ratio (OR) of development of thrombosis in patients with central inserted central catheters (CICCs). (**B**) Forest plot(B1,RE)and funnel plot (B2,RE) showing 15 studies of CICCs with pharmacological deep vein thrombosis prophylaxis data, stratified by anticoagulant drugs. Forest plot showing risk ratio (RR) of development of thrombosis in patients with central inserted central catheters (CICCs). (**C**) Forest plot (C1) and funnel plot (C2) showing 15 studies of CICCs with pharmacological deep vein thrombosis prophylaxis data, stratified by patients populations. Forest plot showing odds ratio (OR) of development of thrombosis in patients with central inserted central catheters (CICCs). PICC = peripherally inserted central catheter; CICC = central inserted central catheter; FE= fixed effect; RE = random effect; OR = odds ratio; RR = risk ratio; VTE = venous thromboembolism.

15 studies of CICCs with pharmacological deep vein thrombosis prophylaxis data were taken to make further stratification analysis about thrombosis by anticoagulant drugs, divided into warfarin group [24, 25, 30, 31, 39, 43, 50, 60], heparin group [37, 40, 42, 44, 49, 60], and the other thrombolytic group [46, 49]. The random effect model was tried firstly to deal with the raw data. The forest plot could be seen in Figure 4, and the overall outcome of the analysis were summarized at the bottom of it (OR = 0.67, 95% CI:0.48–0.93, *p* = 0.02, I^2^ = 57%,Tau^2^ = 0.24, Figure 4A1). Moderate heterogeneity was noted across studies (I^2^ = 51%, *p* = 0.02). Harbord’s test statistic did not suggest obvious publication bias in funnel plot (Figure 4A2). The Newcastle–Ottawa scale was adopted to evaluate study quality and risk of bias in both comparison and non-comparison studies. Studies with a comparison group were considered as high quality. The subgroup of the other thrombolytic group showed better results in heterogeneity than the other two (OR = 0.27, 95% CI:0.15–0.47, *p* < 0.0001, I^2^ = 0%,Tau^2^ = 0.00, Figure 4A1) [46, 49]. We did not take the fixed model to make further analysis for the existence of heterogeneity. The similar results could be seen when we used a random effect model to assess risk ratio of the data (RR = 0.72, 95% CI:0.56–0.93, Z = 2.48, *p* = 0.01, I^2^ = 51%, Tau^2^ = 0.24, Figure 4B).

**Figure 5:**
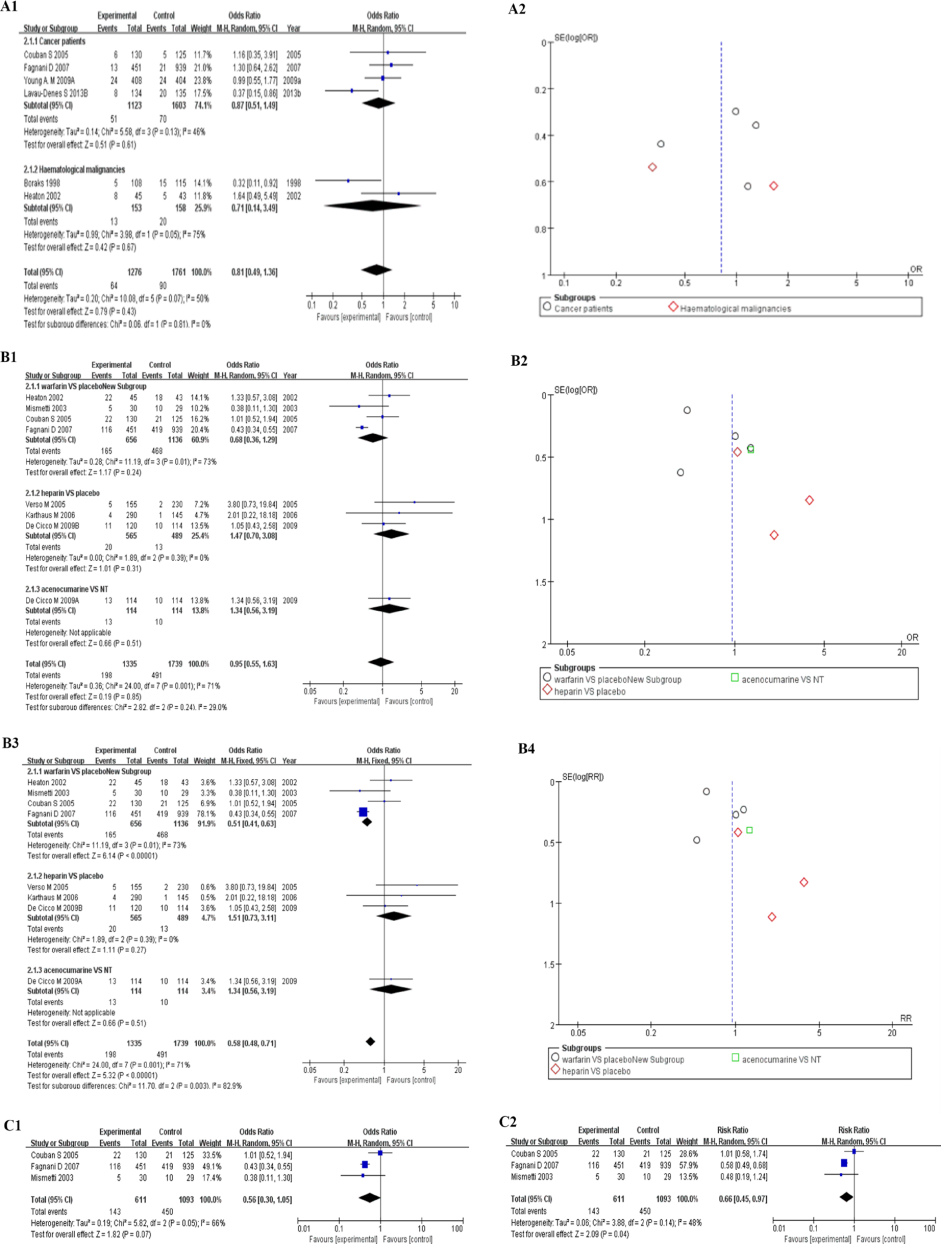
(**A**) Forest plot (A1, RE) and funnel plot (A2, RE) showing 6 of 15 studies with CICCs, stratified by patients populations and pharmacological deep vein thrombosis prophylaxis. Forest plot showing odds ratio (OR) of development of thrombosis in patients with central inserted central catheters (CICCs), stratified by patients populations and pharmacological deep vein thrombosis prophylaxis. (**B**) Forest plot (B1,RE; B3,FE) and funnel plot (B2,RE; B4,FE) showing 8 studies of 2639 patients with CICCs, stratified by pharmacological deep vein thrombosis prophylaxis. Forest plot showing odds ratio (OR) of development of mortality rate in patients with central inserted central catheters (CICCs). (**C**) Forest plot (C1, C2) showing 3 studies with CICCs, stratified by the same pharmacological deep vein thrombosis prophylaxis -- warfarin. PICC = peripherally inserted central catheter; CICC = central inserted central catheter; FE = fixed effect; RE = random effect; OR = odds ratio; RR = risk ratio; VTE = venous thromboembolism.

Then, we divided these 15 studies into another 3 parts by disease kind, including cancer patients [31, 39, 40, 42, 47, 49, 50, 60], haematological malignancies [24, 30, 44, 46], and a mixture of the two [25, 37]. The results revealed obvious heterogeneity among all studies (OR = 0.67, 95% CI:0.48–0.93, Z = 2.41(*p* = 0.02), I^2^ = 57%,Tau^2^ = 0.24, Figure 4C) with the same analysis model (RE) as before. We put further stratification analysis by the same disease and anticoagulant drugs, which were divided into two groups, in order to make sure the reason for heterogeneity [24, 30, 39, 47, 50, 60]. Though, Harbord’s test statistic did not suggest obvious publication bias in funnel plot, the heterogeneity is moderate among studies (OR = 0.81, 95% CI:0.49–1.36, Z = 0.79, *p* = 0.43, I^2^ = 50%, Tau^2^ = 0.20, Figure 5A), especially in haematological malignancies (OR = 0.71, 95% CI:0.14-3.49, Z = 0.42, *p* = 0.67, I^2^ = 75%, Tau^2^ = 0.99, Figure 5A1) [24, 30].

Meta-analysis of 8 studies including 2639 patients, stratified by anticoagulant drugs, showed that pharmacological deep vein thrombosis prophylaxis drugs did not have obvious effects on decreasing the mortality rates of patients with CICCs (OR = 0.95, 95% CI:0.55-1.63, Z = 0.19, *p* = 0.85, I^2^ = 75%, Tau^2^ = 0.36, RE, Figure 5B1 and Figure 5B2) [30, 31, 39, 40, 42, 47, 49]. The heterogeneity is obvious in the subgroup of warfarin [30, 31, 39, 47]. Then, we took 3 studies to make further stratification meta-analysis about the mortality rates [31, 39, 47]. The stratification result of 3 studies showed that warfarin were associated with a decrease on the risk of mortality rate of patients with CICCs (RR = 0.66, 95% CI:0.45–0.97, Z = 2.09(*p* = 0.04); Figure 5C2), while OR of them was of no statistical significance (OR = 0.56, 95% CI:0.30-1.05, Z = 1.82(*p* = 0.07), I^2^ = 66%, Figure 5C1).

## DISCUSSION

The deep vein thrombosis related to indwelling devices such as PICC and CICC is common, especially in malignant disease [69, 70]. There are a lot of reports about the relationship between PICCs and venous thromboembolism, but the incidence and risk of it is unclear [71, 72]. It has been confirmed that venous thromboembolism is a bad signal for the prognosis of malignant disease by plenty of evidence, which is most common in cancer patients [73, 74]. Thrombosis can be caused by a lot of factors, such as tumor itself, anti-tumor therapy and indwelling devices [69, 75]. The understanding of risk about central venous catheter-related side effects is still an important safety question to be faced up, especially for cancer patients as they are prone to be associated with a higher risk of deep vein thrombosis than others [9]. We have not found a systematic review done to deal with these problems just limited to malignant neoplasms. Therefore, we carried out the systematic review and meta-analysis to further investigate this risk in malignant disease patients and tried to reveal the relationship between central venous catheter-related side effects and malignant neoplasms.

In our meta-analysis, 23 included studies about PICC without a comparison group (*n* = 5824) were just displayed by a forest plot showing the pooled, unweighted frequency of patients with peripherally inserted central catheter venous thromboembolism (VTE), which can be seen in (Figure 2) [21, 23, 26–29, 33–36, 38, 41, 45, 47, 52–56, 58, 59, 63, 68]. For these studies, the unweighted frequency of vein thrombosis was 9.2% (536/5824), which is higher than the former reported result 4.48% (189/4223) related to non-tumor and tumor mixed populations [69]. The result of it reveals that PICC-related vein thrombosis is much more prevalent in malignant tumor patients than other populations. It means that the use of PICC in malignant tumor patients would cause a higher risk of developing thrombotic disease.

Though, all kinds of reasons for PICC-related thrombosis have been proposed, such as differences in the anatomical approach to the superior vena cava and more frequent mechanical trauma to the vessel intima in right-handed people, Catheter implantation is the most important causative factor for it [3, 76]. It would be more likely to cause venous obstruction, when indwelling devices are inserted into peripheral veins. However, if PICCs or CICCs are implanted into larger vessels, the incidence of vein thrombosis would be significantly reduced [55]. Tran H reported that PICCs implanted in the internal jugular but arm veins would appeared with a lower incidence of deep vein thrombosis, which supported the suggestion that intimal injury could be associated with PICC-related deep vein thrombosis [55].

Compared to PICCs, CICCs are usually inserted into larger vessels with a lower incidence of vein thrombosis [77]. 7 studies (*n* = 2872) reported PICC-related versus CICC or Port-related vein thrombosis outcomes in malignant tumor patients, which were divided into 3 parts for stratification analysis, listed as cancer patients [22, 57, 61, 67], hematological malignancies [51, 66], and hemato-oncology patients [62]. The result of the stratification analysis could be seen in (Figure 3), which supported the theory that CICCs were associated with a decrease in the odds ratio of thrombosis compared with PICCs (OR = 0.45, 95% CI:0.32–0.62, *p* < 0.0001, I^2^ = 0%,Tau^2^ = 0.00; Figure 3A), especially in cancer patients (OR = 0.30, 95% CI:0.12–0.75, *p* < 0.05, I^2^ = 0%, Tau^2^ = 0.00, Figure 3A1) [22, 57, 61, 67], agreed with the former research [77]. Similar outcome could also be seen in (Figure 3B), calculated by a fixed effect model. All the results were of statistical significance without obvious heterogeneity. Above the results, we concluded that if the patients appeared with the risk of deep vein thrombosis before catheter insertion, CICC may be a better choice for patients with malignant neoplasms, especially for cancer patients.

As for the heightened risk of deep vein thrombosis, should PICC recipients routinely receive pharmacological DVT prophylaxis in malignant neoplasms? Although more and more clinical trials were put into practice in cancer-related thrombosis [78], little suitable data for our meta-analysis could be collected in PICC-related area. Instead, 15 studies of CICCs with pharmacological deep vein thrombosis prophylaxis data were collected to make further stratification analysis about thrombosis by anticoagulant drugs, divided into warfarin group [24, 25, 30, 31, 39, 43, 50, 60], heparin group [37, 40, 42, 44, 49, 60], and other thrombolytic group [46, 49]. Among these studies, warfarin is the most commonly used anticoagulant drug in patients with CICCs [24, 25, 30, 31, 39, 43, 50, 60]. We could get the conclusion that anticoagulant drug is a beneficial factor in decreasing the incidence rate of thrombosis. The details of the meta-analysis results were gathered in (Figure 4A) and (Figure 4B), including forest and funnel plot. All the anticoagulant drugs could also been found in other researches with the risk of bleeding among cancer patients [79]. We did not take the risk of bleeding for further meta-analysis, because it is not the main object of our study. Furthermore, we did not take the fixed effect model to make further analysis for the existence of heterogeneity.

When these 15 studies were divided into another 3 parts by disease kind, such as cancer patients [31, 39, 40, 42, 47, 49, 50, 60], haematological malignancies [24, 30, 44, 46], and a mixture of the two [25, 37], the result of the meta-analysis was of significance (OR = 0.67, 95% CI:0.48-0.93, Z = 2.41(*p* = 0.02), I^2^ = 57%, Tau^2^ = 0.24, RE, Figure 4C), while obvious heterogeneity among all studies still existed (OR = 0.81, 95% CI:0.49–1.36, Z = 2.38(*p* = 0.02), I^2^ = 50%,Tau^2^ = 0.20, RE, Figure 5A), especially in haematological malignancies (OR = 0.71, 95% CI:0.14–3.49, Z = 0.42, *p* = 0.67, I^2^ = 75%, Tau^2^=0.99, Figure 5A1) [24, 30]. We could conclude from (Figure 4) that anticoagulant drug is a beneficial factor in decreasing the incidence rate of thrombosis of patients with CICCs.

Meta-analysis of 8 studies involving 2639 patients showed that pharmacological deep vein thrombosis prophylaxis drugs could not decrease the risk of mortality (OR = 0.95, 95% CI:0.55–1.63, Z = 0.19, *p* = 0.85, I^2^ = 75%, Tau^2^ = 0.36, RE, Figure 5B1, Figure 5B2) of malignant tumor patients with CICCs [30, 31, 39, 40, 42, 47, 49]. The heterogeneity is obvious in the subgroup of warfarin [30, 31, 39, 47]. Therefore, we did not take the result of the fixed effect model into account (OR= 0.58, 95% CI:0.48–0.71, Z = 5.32, *p* < 0.0001, I^2^ = 71%, FE, Figure 5B3, Figure 5B4). Compared to the former meta-analysis, it is the first time for the relationship between pharmacological deep vein thrombosis prophylaxis drugs and the mortality risk of malignant tumor patients with CICCs to be revealed by us [9]. Then, we took 3 studies to make further stratification meta-analysis about the mortality rates [31, 39, 47]. The stratification result of 3 studies supported the suggestion that warfarin was associated with a decrease on mortality rate of cancer patients with CICCs (RR = 0.66, 95% CI:0.45–0.97, Z = 2.09(*p* = 0.04); Figure 5C2), while OR of them was of no statistical significance (OR = 0.56, 95% CI:0.30–1.05, Z = 1.82(*p* = 0.07), I^2^ = 66%, Figure 5C1).

In conclusion, we found that PICCs are associated with a higher risk of deep vein thrombosis, when compared with CICCs. We could get the conclusion that pharmacological deep vein thrombosis prophylaxis drugs is a beneficial factor in decreasing the incidence of thrombosis and warfarin may decrease the risk of mortality of malignant tumor patients with CICCs.

## MATERIALS AND METHODS

### Search strategy and selection criteria

We took the method called the Preferred Reporting Items for Systematic Reviews and Meta-Analyses (PRISMA) to search materials [10]. During the process of searching, we mainly pay our attention to English studies ranged from Jan 01,1970 to June 30,2017 (key words: “Cancers”, “Central catheters”, “peripherally inserted central catheter”, “PICC”, “central inserted central catheters ”, “CICC”, “CVC”, “deep vein thrombosis”, “pulmonary embolism”, “venous thromboembolism”, “death”, “mortality”). Our review includes both independent and industry sponsored studies. We collected the studies in human beings which were presented in full text, abstract, or poster form. The searching history of PubMed was listed in (supplementary material). The Conference Papers Index which was provided by ProQuest (1982–2017), Biosis (1926–2017), and Scopus (1996–2017), was used to collate conference posters and abstracts. Some ongoing clinical trials were confirmed from American or European clinical trial centers, and other data of interest were gathered from information seeking on the internet and manual access of bibliographies. We had selected four authors independently to confirm their eligibility, and then get agreement together.

Subjects enrolled in the study must meet the following criteria: (1) case–control studies about the relevance between Central catheters and cancer patients; (2) available data in cases and controls provided; (3) self-reported results and risk assessment and/or displayed data necessary for evaluating OR with 95% CI or other evaluable indicators such as RR, HR and so on; (4) included participants 18 years of age or older. Exclusion criteria: (1) studies that crossing with other studies or reported with data from the same authors; (2) studies involved neonates or patients younger than 18 years; (3) complications not related to the purpose of the study; (4) thrombophlebitis but not venous thrombosis; (4) PICC through the leg implanted but the arm; (5) case report about unusual complications.

### Data extraction and validity assessment

The extraction of the data was carried out in accordance with the criteria recommended by the Cochrane Collaboration [11]. Treatment groups were confirmed as patients who had PICC or CICC implanted for any indication. We collected the information of the study including the number of patients, population, incidence rate of deep vein thromboses or pulmonary embolisms, indication for central venous duct placement, the position of the central venous duct tip, and use of drugs for prevention of deep vein thrombosis, the survival status of malignant tumor patients. If no useful data was extracted, we would try to get in touch with the author for further information. We divided collected studies into three categories: (1) PICC compared with other infusion methods but CICC; (2) PICC compared with CICC; (3) CICC compared with other infusion methods but PICC.

### Assessment of bias risk

Four authors (including two clinicians, a nurse and a statistical analyst) evaluated the risk of central catheters for malignant tumor patients independently. The study quality was judged by Newcastle-Ottawa scale as proposed by the Cochrance Collaboration [12]. The meta-analyses mainly checked up venous thrombosis (VT) and mortality rates for all studies. We tried our best to estimate for risk ratio (RR), odds ratio (OR), hazard ratios (HR), and 95% confidence interval (CI) which were derived from Review Manager 5.3, calculated with random effect (RE) or fixed effect (FE) models according to the actual situation of the data. If we could not get the data about mortality events, we would take the inverse variance method to calculated HR. when VT events were not available, a correction factor (0.5) was adopted to revise the RR. The results of effect estimates were considered as statistically significant when *P* value is less than 0.05.

### Main outcome measures

We designated independent researchers to collect the information on venous thrombosis (VT) or pulmonary embolism, survival status and comparison. Deep vein thrombosis was defined as thrombosis related to the deep veins of the arm (brachial, axillary, subclavian, or internal jugular veins) which could be diagnosed by compression ultrasonography, venography, X-ray or CT scan. Survival status included the information about mortality rate of malignant tumor patients and complications affecting the prognosis due to implantation of central catheters but VT. If some information was unclear in the included study, we would get in touch with the study authors to make sure whether the detail of the data was available. If the useful detail was unavailable, the study would be precluded from the analysis [13–17]. Where disagreements was found, the corresponding author of the article would deal with the differences.

### Statistical analysis

Data of all enrolled studies were summarized with odds ratio (OR) by using the Comprehensive Meta-Analysis software according to whether or not they featured a comparison group. An OR is supposed to be a more conservative estimate and may be more likely to detect a safety signal, as the method by which an OR is calculated provides a point estimate farther from unity than that provided by a HR. We took a random effect model to evaluate most of treatment effects which are different among all studies [18]. We also used a fixed effect model occasionally for some analysis when the treatment effects were deemed to be the same and that differences in results were just due to random probability. We collected incidence rate of patients with venous thrombosis from the unit, and then gathered them in non-comparison studies, with variance estimates generated from the enhanced arcsine transformation for data with binomial distributions [19]. Cochrane’s *Q* statistic and the *I*^2^ statistic were taken to deal with the heterogeneity among studies just as recommended by Higgins and colleagues [20]. Harbord’s test was used to assess publication bias for studies; *p* values less than 0.05 was deemed to publication bias. We tried to collect all survival data of cancer patients including long-term follow-up data. For chemotherapy studies, an influence plot was generated that shows the estimated OR for mortality if an individual chemotherapy study was precluded from the analysis. All data consolidation and analyses were carried out by Review Manager 5.3. Statistical tests were all two-sided. Effect estimates were deemed statistically significant when *p* value ≤ 0.05.

## SUPPLEMENTARY MATERIALS TABLES









## Abbreviations

PICC: peripherally inserted central catheter
CICC: central inserted central catheter
OR: odds ratio
RR: risk ratio
VTE: venous thromboembolism
RE: random effect
FE: fixed effect
VT: venous thrombosis
HR: hazard ratio
CI: confidence interval
