# Supplementary material for: Risk associated with central catheters for malignant tumor patients: a systematic review and meta-analysis
Source: Oncotarget. 2018 Jan 12;9(15):12376–88. doi: 10.18632/oncotarget.24212 (PMC5844754; doi:10.18632/oncotarget.24212)
Supplement: Supplementary file 1 [file oncotarget-09-12376-s001.pdf]

Risk associated with central catheters for malignant tumor patients: a systematic review and meta-analysis

SUPPLEMENTARY MATERIALS

Supplementary Table 1: PRISMA of the manuscript. See Supplementary Table 1

Supplementary Table 2: The searching history of pubmed. See Supplementary Table 2

Supplementary Table 3: Characteristics of included studies. See Supplementary Table 3
