# Supplementary material for: Risk associated with central catheters for malignant tumor patients: a systematic review and meta-analysis
Source: Oncotarget. 2018 Jan 12;9(15):12376–88. doi: 10.18632/oncotarget.24212 (PMC5844754; doi:10.18632/oncotarget.24212)
Supplement: Supplementary file 3 [file oncotarget-09-12376-s003.doc]

**Recent queries in pubmed**

**Search,Query,Items found,Time**

#73,"Search (#40) AND #53 Filters: Clinical Study; Clinical Conference; Classical Article; Clinical Trial; Comparative Study; Controlled Clinical Trial; Evaluation Studies; Multicenter Study; Observational Study; Pragmatic Clinical Trial; Randomized Controlled Trial; Twin Study; Validation Studies; Abstract; Publication date to 2017/06/30; Humans",137,11:31:58

#72,"Search (#40) AND #53 Filters: Clinical Study; Clinical Conference; Classical Article; Clinical Trial; Comparative Study; Controlled Clinical Trial; Evaluation Studies; Multicenter Study; Observational Study; Pragmatic Clinical Trial; Randomized Controlled Trial; Twin Study; Validation Studies; Abstract; Humans",137,11:28:06

#71,"Search (#40) AND #53 Filters: Clinical Study; Clinical Conference; Classical Article; Clinical Trial; Comparative Study; Controlled Clinical Trial; Evaluation Studies; Multicenter Study; Observational Study; Pragmatic Clinical Trial; Randomized Controlled Trial; Twin Study; Validation Studies; Abstract",137,11:27:57

#70,"Search (#40) AND #53 Filters: Clinical Study; Clinical Conference; Classical Article; Clinical Trial; Comparative Study; Controlled Clinical Trial; Evaluation Studies; Multicenter Study; Observational Study; Pragmatic Clinical Trial; Randomized Controlled Trial; Twin Study; Validation Studies",140,11:27:50

#69,"Search (#40) AND #53 Filters: Clinical Study; Clinical Conference; Classical Article; Clinical Trial; Comparative Study; Controlled Clinical Trial; Evaluation Studies; Multicenter Study; Observational Study; Pragmatic Clinical Trial; Randomized Controlled Trial; Twin Study",140,11:27:47

#68,"Search (#40) AND #53 Filters: Clinical Study; Clinical Conference; Classical Article; Clinical Trial; Comparative Study; Controlled Clinical Trial; Evaluation Studies; Multicenter Study; Observational Study; Pragmatic Clinical Trial; Randomized Controlled Trial",140,11:27:43

#67,"Search (#40) AND #53 Filters: Clinical Study; Clinical Conference; Classical Article; Clinical Trial; Comparative Study; Controlled Clinical Trial; Evaluation Studies; Multicenter Study; Observational Study; Pragmatic Clinical Trial",140,11:27:39

#66,"Search (#40) AND #53 Filters: Clinical Study; Clinical Conference; Classical Article; Clinical Trial; Comparative Study; Controlled Clinical Trial; Evaluation Studies; Multicenter Study; Observational Study",140,11:27:36

#65,"Search (#40) AND #53 Filters: Clinical Study; Clinical Conference; Classical Article; Clinical Trial; Comparative Study; Controlled Clinical Trial; Evaluation Studies; Multicenter Study",140,11:27:33

#64,"Search (#40) AND #53 Filters: Clinical Study; Clinical Conference; Classical Article; Clinical Trial; Comparative Study; Controlled Clinical Trial; Evaluation Studies",134,11:27:29

#63,"Search (#40) AND #53 Filters: Clinical Study; Clinical Conference; Classical Article; Clinical Trial; Comparative Study; Controlled Clinical Trial",130,11:27:25

#62,"Search (#40) AND #53 Filters: Clinical Study; Clinical Conference; Classical Article; Clinical Trial; Comparative Study",130,11:27:21

#61,"Search (#40) AND #53 Filters: Clinical Study; Clinical Conference; Classical Article; Clinical Trial",80,11:27:18

#60,"Search (#40) AND #53 Filters: Clinical Study; Clinical Conference; Classical Article",80,11:27:13

#59,"Search (#40) AND #53 Filters: Clinical Study; Clinical Conference",80,11:27:09

#58,"Search (#40) AND #53 Filters: Clinical Study",80,11:27:03

#54,"Search (#40) AND #53",697,11:27:00

#53,"Search (((((((((((#41) OR #42) OR #43) OR #44) OR #45) OR #46) OR #47) OR #48) OR #49) OR #50) OR #51) OR #52",4836842,11:22:36

#52,"Search death risk",169634,11:19:49

#51,"Search mortality risk",334334,11:19:25

#50,"Search death rate",1082662,11:19:12

#49,"Search mortality rate",1039009,11:19:04

#48,"Search livability",245,11:18:35

#47,"Search Death",717174,11:18:15

#46,"Search mortality",1039009,11:17:57

#45,"Search survival",1635075,11:17:36

#44,"Search prognositic",24,11:17:14

#43,"Search safety",474836,11:16:58

#42,"Search risk",2083484,11:15:57

#41,"Search Prognosis",1489092,11:15:42

#40,"Search (#24) AND #39",1164,11:22:35

#39,"Search ((((((((((((#25) OR #26) OR #27) OR #28) OR #29) OR #30) OR #32) OR #33) OR #34) OR #35) OR #36) OR #37) OR #38",578348,11:14:30

#38,"Search venous thromboembolism",21251,11:11:50

#37,"Search pulmonary embolism",48945,11:09:52

#36,"Search vein thrombosis",87489,11:09:25

#35,"Search deep vein thrombosis",77716,11:09:10

#34,"Search Embolism",86964,11:08:54

#33,"Search Thrombosis",194569,11:07:25

#32,"Search Thromboembolism",66561,11:07:09

#31,"Search neuropathy",62716,11:06:41

#30,"Search Neopathy",2,11:06:40

#29,"Search untoward effect",1376,11:05:58

#27,"Search adeverse reaction",0,11:04:51

#28,"Search adverse reaction",67323,11:04:51

#26,"Search side reaction",28765,11:04:34

#25,"Search complication",233910,11:04:14

#24,"Search (#12) AND #23",2856,11:03:47

#23,"Search (((((((#13) OR #14) OR #15) OR #16) OR #17) OR #18) OR #19) OR #22",21168,11:03:21

#22,"Search CVCs",1147,11:01:37

#19,"Search CVC",3584,11:01:20

#18,"Search PICCs",527,11:01:08

#17,"Search PICC",840,11:00:55

#16,"Search venous catheters",17033,11:00:42

#15,"Search central venous catheters",10563,11:00:30

#14,"Search peripherally inserted central catheters",911,11:00:15

#13,"Search central catheters",12406,10:59:54

#12,"Search ((((((((((#1) OR #2) OR #3) OR #4) OR #5) OR #6) OR #7) OR #8) OR #9) OR #10) OR #11",3935272,10:59:28

#11,"Search carcinoma",831887,10:57:47

#10,"Search pre-malignant",1654,10:57:31

#9,"Search premalignant",47284,10:57:14

#8,"Search malignant",334048,10:57:00

#7,"Search tumor",3428485,10:56:43

#6,"Search tumour",3428485,10:56:33

#5,"Search precancer",1451,10:55:42

#4,"Search pre-cancer",377,10:55:23

#3,"Search cancers",2989241,10:55:03

#2,"Search cancer",3502551,10:53:48

#1,"Search neoplasm[MeSH Terms]",2924997,10:52:10
