# Supplementary material for: Risk associated with central catheters for malignant tumor patients: a systematic review and meta-analysis
Source: Oncotarget. 2018 Jan 12;9(15):12376–88. doi: 10.18632/oncotarget.24212 (PMC5844754; doi:10.18632/oncotarget.24212)
Supplement: Supplementary file 4 [file oncotarget-09-12376-s004.doc]

| **Supplementary Table 3: Characteristics of included studies** | | | | | | | | |
| --- | --- | --- | --- | --- | --- | --- | --- | --- |
| Bottino J. (1979)^21^ | Prospective | Hematological malignancies and solid tumors | PICC | NR | Dressings were changed 3 times per week, with sterile gauze and povidone-iodine ointment placed over the entry site | 87 | 1 | Full text |
| Carde P. (1989)^22^ | Prospective, a randomized trial | Solid Tumors | CE VS TI | NR | NR | 96 | 3 | Abstract |
| Nightingale C. E. (1997)^23^ | Prospective | Gastrointestinal Malignancy | CVC | All patients received 1 mg warfarin daily as prophylaxis against catheter thrombosis. | The unused lumens were flushed twice weekly with heparinised saline via a rubber luer lock cap which was replaced every 4 weeks. | 817 | 42 | Full text |
| Boraks Paul. (1998) A^24^ | Retrospective | Haematological Malignancies | CVC | Placebo | Heparin flush twice weekly | 115 | 15 | Full text |
| Boraks Paul. (1998) B^24^ | Retrospective | Haematological Malignancies | CVC | Warfarin 1 mg/day | Heparin flush twice weekly | 108 | 5 | Full text |
| Ratcliffe M. (1999)^25^ | Prospective | Solid or Haematological Malignancy | CVC | warfarin 1 mg/day VS Placebo | Daily heparin–saline flushes | 84 | 10 | Full text |
| Mukherjee. (2001)^26^ | Retrospective | Metastatic Colorectal Cancer Patients | CVC | No | NR | 385 | 20 | Conference  abstract |
| Strahelivitz. (2001)^27^ | Retrospective | Acute Myeloid Leukemia | PICC | NR | NR | 40 | 2 | Full text |
| Walshe. (2002)^28^ | A 1-year prospective observational study | Cancer Patients | PICC | NR | NR | 335 | 12 | Full text |
| Fijnheer R. (2002)^29^ | Prospective | Haematologic Malignancy | CVC | Daily Heparin-Saline Flushes | Flushed with heparin-saline (100 IU/ml). | 94 | 9 | Full text |
| Heaton D. C. (2002)^30^ | Prospective | Haematological Malignancies | CVC | warfarin VS placebo | All catheters were flushed twice daily with 50 μg heparin (Hickman) or saline (Groshong). | 88 | 13 | Full text |
| Mismetti P. (2003)^31^ | A prospective, randomized, open, parallel-group, multicenter trial | Nonhematologic cancer Patients | CVC | Warfarin VS nadroparin | The catheter lumen was flushed with 10 mL of saline solution and 5 mL of heparinized saline solution (500 IU of heparin) after catheter insertion, after each blood collection, after each infusion chemotherapy, and otherwise at least once a week | 45 | 10 | Full  text |
| Cortelezzi A. (2003)^32^ | Retrospective | Hematological Malignancies | CVC | Weekly heparin flush and LMWH or UFH or none | Heparin (UFH), 2,500 IU daily infusion in 169 CVCs, or low molecular weight heparin (LMWH), 3,800 IU daily | 137 | 14 | Abstract |
| Lin and Walker. (2004)^33^ | Retrospective | Cancer patients | PICC | NR | NR | 190 | 9 | Conference  abstract |
| Chu SY. (2004)^34^ | Retrospective | Acute leukemia | PICC | NR | NR | 41 | 3 | Conference  abstract |
| Cortelezzi A. (2005)^35^ | Prospective, observational and multicentre study | Hematological malignancies | CVC(65.9 %),PICC(2.8%), port (1.7%) | Antiplatelet drugs were considered antithrombotic prophylaxis | Daily dosages >5000 UI of unfractionated heparin, low molecular weight heparins | 458 | 35 | Full text |
| Abdullah BJJ. (2005)^36^ | Prospective | Cancer Patients | PICC | NR | Low dose heparin but AML patients | 26 | 10 | Full text |
| Abdelkefi A. (2005 Nov)^37^ | A randomized, controlled,prospectively trial | Hematologic and Oncologic Disease | CVC | Heparin VS normal saline solution | Intravenous unfractionated heparin (continuous infusion of 100 U/kg per day) or 50 mL/day of normal saline solution as a continuous infusion (control group). | 204 | 12 | Full text |
| Rose M. (2005)^38^ | Retrospective | Head and neck cancer | PICC | NR | NR | 36 | 4 | Conference  abstract |
| Couban S. (2005)^39^ | Randomized Placebo-Controlled Study | Cancer patients | CVC | warfarin VS placebo | The use of antiplatelet medication or low-dose standard heparin or low molecular weight heparin as veno-occlusive disease prophylaxis | 325 | 6 | Full text |
| Verso M. (2005)^40^ | Prospective | Cancer patients | CVC | Enoxaparin VS Placebo | Enoxaparin started 2 hours before CVC insertion and continued for 6 weeks. | 385 | 50 | Full text |
| Lee AY. (2006)^41^ | Prospective | Cancer patients | CVC | NR | NR | 44 | 15 | Full text |
| Karthaus M. (2006)^42^ | A double-blind, placebo-controlled phase III trial | Cancer patients | CVC | Dalteparin VS Placebo | Dalteparin once daily for 16 weeks. | 439 | 16 | Full text |
| Fagnani D. (2007)^43^ | A observational prospective study | Cancer patients | CVC | Warfarin VS Placebo | NR | 1390 | 34 | Full text |
| Niers T. M. (2007)^44^ | A single-center, prospective, randomized, placebo-controlled double-blind trial | Hematologic malignancies | CVC | Nadroparin VS Placebo | Once-daily nadroparin injection | 107 | 11 | Full text |
| Simcock L.(2008)^45^ | Retrospective | Cancer patients | PICC | NR | NR | 375 | 33 | Abstract |
| van Rooden C. J. (2008)^46^ | A  randomized double-blind controlled trial | Hematologic malignancies | CVC | Urokinase rinses VS Placebo (saline) | urokinase rinses (5 mL of 5,000 U/mL) three times weekly | 160 | 13 | Full text |
| Derudas D. (2009)^47^ | Retrospective | Hematological malignancies | PICC | NR | NR | 96 | 3 | Conference  abstract |
| Calderero Aragon V. (2009)^48^ | Prospective | Oncological patients | CVC | Low molecular weight heparins (bemiparin) | Once-daily heparin infusion | 148 | 11 | Abstract |
| De Cicco M. (2009)A^49^ | Prospective,a randomized controlled study | Cancer patients | CVC | Acenocumarine VS NT | Acenocumarine1 mg/day for 3 days before and 8 days after CVC insertion (group A) or dalteparin 5000 IU 2 h before and daily for 8 days after CVC insertion (group D) or no anticoagulant treatment (group NT). | 228 | 85 | Full text |
| De Cicco M. (2009)B^49^ | Prospective,a randomized controlled study | Cancer patients | CVC | Aalteparin VS NT | Acenocumarine1 mg/day for 3 days before and 8 days after CVC insertion (group A) or dalteparin 5000 IU 2 h before and daily for 8 days after CVC insertion (group D) or no anticoagulant treatment (group NT). | 234 | 108 | Full text |
| Young A. M. (2009)A^50^ | Prospective, randomly assigned | Cancer patients | CVC | Fixed-dose warfarin 1 mg per day vs No | Fixed-dose warfarin 1 mg per day | 812 | 48 | Full text |
| Young A. M. (2009)B^50^ | Prospective, randomly assigned | Cancer patients | CVC | Dose-adjusted warfarin per day to maintain an international normalised ratio between 1.5 and 2.0 VS No | Dose-adjusted warfarin per day to maintain an international normalised ratio between 1.5 and 2.0 | 944 | 47 | Full text |
| Worth L. J. (2009)^51^ | Prospective | Hematological Malignancy | CVC VS PICC | Heparin | When not in use, a heparin lock (heparinized saline, 50U in 5mL) was instilled weekly into each CVC. | 106 | 16 | Full text |
| Romagnoli E. (2010)^52^ | Retrospective | Cancer petients | PICC | NR | NR | 49 | 3 | Conference  abstract |
| Pari C. (2010)^53^ | Prospective | Cancer petients | PICC | NR | NR | 70 | 1 | Conference  abstract |
| Yue ZY. (2010)^54^ | Retrospective | Cancer petients | PICC | NR | NR | 400 | 8 | Abstract |
| Tran H. (2010)^55^ | 窗体顶端  Retrospective | Hematological malignancies | PICC | No | NR | 498 | 39 | Full text |
| Ahn DH. (2011)^56^ | Retrospective | Cancer Patients | PICC | NR | NR | 237 | 36 | Conference  abstract |
| Catalano O. (2011)^57^ | Prospective | Oncologic patients | Ports VS PICC | NR | NR | 479 | 17 | Full text |
| Xing L. (2012)^58^ | Retrospective | 窗体顶端 Breast cancer patients | PICC | NR | NR | 187 | 4 | Full text |
| Aw A. (2012)^59^ | A retrospective cohort study | Cancer patients | PICC | No | NR | 340 | 19 | Full text |
| Lavau-Denes S.(2013)A^60^ | A randomized, controlled, phase III study | Cancer patients | CVC | Subcutaneous Low Molecular Weight Heparin (LMWH); | NR | 273 | 34 | Full text |
| Lavau-Denes S. (2013)B^60^ | A randomized, controlled, phase III study | Cancer patients | CVC | Oral warfarin 1 mg/day | NR | 269 | 28 | Full text |
| Patel G. S. (2014)^61^ | Prospective | Non-haematological malignancies | CVC VS PICC | NR | Weekly flushing of catheters with saline and once weekly redressing of catheter exit site using an aseptic technique | 70 | 4 | Full text |
| Sriskandarajah P.(2015)^62^ | Retrospective, single center cohort analysis | Hemato-oncology patients | LTSTC vs PICC | NR | NR | 583 | 24 | Full text |
| Yuxiu Liu. (2015)^63^ | A double-center prospective investigation | Cancer patients | PICC | NR | Flushed with 10 mL saline after placement, after each use, and once a week between chemotherapy. | 311 | 160 | Full text |
| Bertoglio S. (2016)^64^ | A prospective study | Non-haematological malignancies | PICC | NR | Flushing and locking of PICCs with prefilled 10 ml normal saline syringes by pulsatile method before and after every i.v. drug delivery | 291 | 34 | Full text |
| Luo L. (2016)^65^ | A Prospective Study | Oncology Patients | PICC | Low-molecular-weight heparin | Low-molecular-weight heparin therapy was initiated once thrombosis was identified by Doppler sonography | 246 | 90 | Full text |
| Mohammad Refaei. (2016)^66^ | A comparative, retrospective study | Acute leukemia patients | CVC VS PICC | NR | NR | 1331 | 123 | Full text |
| Shirong Fang. (2017) A^67^ | A double-center prospective cohort study | Cancer Patients | CVC VS PICC | NR | Normal saline were used to flush the catheter.After completion of the infusion therapy, 10 mL heparin saline (100 IU/mL) was used to seal the catheter. | 100 | 10 | Full text |
| Shirong Fang.(2017) B^67^ | A double-center prospective cohort study | Cancer Patients | Port VS PICC | NR | The port should be washed every 4 weeks.After completion of the infusion therapy, 10 mL heparin saline (100 IU/mL) was used to seal the catheter. | 105 | 8 | Full text |
| Daniel Jones. (2017)^68^ | Retrospective | Cancer Patients | PICC | No routine use of thromboprophylaxis | NR | 490 | 27 | Full text |

**PICC** = peripherally inserted central catheter. **VTE** = venous thromboembolism. **DVT** = deep vein thrombosis. **NR** = No reported. **CVC=** central venous catheters. **CICC** = central inserted central catheter.
